# The effects of trans-resveratrol on insulin resistance, inflammation, and microbiota in men with the metabolic syndrome: A pilot randomized, placebo-controlled clinical trial

**Published:** 2018-12-07

**Authors:** Jeanne M. Walker, Patricia Eckardt, Jose O. Aleman, Joel Correa da Rosa, Yupu Liang, Tadasu Iizumi, Stephane Etheve, Martin J. Blaser, Jan L.Breslow, Peter R. Holt

**Affiliations:** ^1^The Rockefeller University Hospital, New York; ^2^Laboratory of Biochemical Genetics and Metabolism, Rockefeller University, New York; ^3^Department of Medicine, New York University School of Medicine, New York; ^4^DNP R&D Analytics, DSM Nutritional Products LTD, Kaiseraugst, Switzerland

**Keywords:** metabolic syndrome, euglycemic hyperinsulinemic clamp, insulin resistance, resveratrol, dihydroresveratrol, fecal microbiota, *akkermansia muciniphila*, adipose tissue gene expression

## Abstract

**Background and Aim:**

The metabolic syndrome (MetS) is a pathological condition comprised of abdominal obesity, insulin resistance, hypertension, and hyperlipidemia. It has become a major threat globally, resulting in rapidly increasing rates of diabetes, coronary heart disease, and stroke. The polyphenol resveratrol (RES) is believed to improve glucose homeostasis and insulin resistance by activating sirtuin, which acetylates and coactivates downstream targets and affects glucose and lipid homeostasis in the liver, insulin secretion in the pancreas, and glucose uptake in skeletal muscle. We studied the effects of RES on insulin resistance, glucose homeostasis, and concomitant effects on adipose tissue metabolism and fecal microbiota in insulin-resistant subjects with the MetS.

**Methods:**

A total of 28 obese men with the MetS were studied during a 35-day stay in the Rockefeller University Hospital metabolic unit. Subjects were randomized to receive RES 1 g orally twice daily or placebo while kept weight stable and consuming a western-style diet. At baseline, and after 30 days of RES or placebo administration, subjects underwent testing that included a euglycemic, hyperinsulinemic clamp, 2-h oral glucose tolerance test (GTT), resting energy expenditure, daily blood pressure monitoring, abdominal adipose tissue biopsy, and fecal and blood collections.

**Results:**

RES induced no changes in insulin resistance but reduced the 120-min time point and the area under the curve for glucose concentration in the 2-h GTT. In *post-hoc* analysis, Caucasian subjects showed a significant improvement in insulin sensitivity and glucose homeostasis after GTT, whereas non-Caucasians showed no similar effects. Levels of fasting plasma RES and its primary metabolite dihydroresveratrol were variable and did not explain the racial differences in glucose homeostasis. RES administration to Caucasian subjects leads to an increase in several taxa including *Akkermansia muciniphila*.

**Conclusions:**

RES 2 g administered orally to obese men with MetS and insulin resistance marginally altered glucose homeostasis. However, in a small group of Caucasians, insulin resistance and glucose homeostasis improved. No concomitant changes in adipose tissue metabolism occurred, but fecal microbiota showed RES-induced changes.

**Relevance for Patients:**

The MetS increases the risk of diabetes, heart disease, and stroke. A major component of the syndrome is insulin resistance, resulting in systemic inflammation and hyperinsulinemia. The primary treatment consists of lifestyle changes, improved diet, and increased physical activity. This is often unsuccessful. In this study, RES was well tolerated. In Caucasian men, it significantly improved insulin sensitivity and glucose homeostasis. Similar results were found in studies that consisted exclusively of Caucasian men. However, RES presents a novel addition to the current treatment of the MetS and its sequelae.

## 1. Introduction

The metabolic syndrome (MetS) is defined by central obesity, pre-diabetes, insulin resistance, lipid disorders, and hypertension. MetS is associated with an increased risk for cardiovascular disease, diabetes, and cancer [1]. About one-third of the adult U.S. population has the MetS, and prevalence increases with age [2,3]. Compared to adults 20–39 years of age, those 40–59 years of age were 3 times as likely, and those over the age of 60 were 6 times as likely to have MetS [3]. With aging of the population, the incidence of this condition is expected to rise markedly.

Insulin resistance is a central component of the MetS and is associated with obesity in which the hormone signaling cascade is diminished in adipose tissue and skeletal muscle. The liver fails to decrease glucose production in the presence of hyperglycemia, and lipid stores are released from adipose tissue. This reduction in insulin action results in increased inflammation which contributes further to the insulin resistance [4]. Insulin resistance sets the stage for progression to diabetes by increasing the demand on pancreatic beta cells to produce insulin, eventually exhausting the insulin supply.

In this study, we examine the effects of trans-resveratrol (RES) (3,5,4’-trihydroxy-trans-stilbene), a natural product extracted from Japanese knotweed [5], peanuts, grapes, and certain berries [6], on insulin resistance in obese men with the MetS. RES dietary intake is very low, estimated to be only about 100 µg/day from dietary intake [7]. RES can act as a polyphenolic sirtuin (SIRT1) activator to acetylate and activate or deactivate many substrates, including Forkhead transcription factor and peroxisome proliferator-activated receptor-y by transactivating the coactivator proliferator-activated receptor-γ coactivator 1α (PGC-1α) [8]. This is accompanied by effects on both glucose and lipid metabolism. SIRT1 is expressed highly in many tissues including muscle, pancreas, and adipose tissues. Thus, RES, as a natural activator of SIRT1, has the capacity to greatly affect nutrient metabolism. Fat deposition can be inhibited and pancreatic β cells protected from cytokine-induced damage [9]. Specifically, RES has been reported to improve glucose tolerance and insulin resistance in rodents [10] and humans [11]. In patients with cardiovascular disease, RES has been reported to reduce inflammation, as demonstrated by decreased circulating inflammatory markers such as C-reactive protein, interleukin 1 (IL-1), and IL-6 [12]. In humans, RES was also found to significantly suppress postprandial glucagon responses [13], contributing to improved insulin sensitivity. In a meta-analysis evaluating 11 studies involving 388 diabetic subjects, Liu *et al*. [14] found that RES significantly reduced fasting glucose, insulin, hemoglobin A1C, and insulin resistance as measured by the homeostatic model assessment (HOMA). In patients with cancer, RES decreased oxidative stress and inflammation, through targeting of TANK-binding kinase 1, which is activated in many chronic inflammatory diseases [15].

RES can be modified by microbiota principally present in the colon, leading to the transformation of several metabolic products [16]. Furthermore, in mice, RES can alter the gut microbiota with important metabolic effects, including reducing fat stores, thereby decreasing obesity [17]. RES also can alter the formation of trimethylamine-*N*-oxide (TMAO), through remodeling of the microbiota and inhibition of bile acid synthesis. This reduction of TMAO by RES may decrease the development of atherosclerosis and coronary heart disease [18]. RES administration has been accompanied by changes in subcutaneous and visceral adipose tissues in rodents [19,20].

The primary hypothesis of our study was to determine if high dose RES, administered to obese men with insulin resistance and the MetS, would improve insulin sensitivity, glucose tolerance, and other features of the MetS and thus possibly contribute to established therapies of the disease. Secondary aims were to determine whether RES-induced changes in the MetS were accompanied by concomitant changes in gene expression of adipose tissue and on gut microbiota, as determined in the feces. Previous clinical trials of RES have shown improvement in one of the components of the MetS as discussed above. To our knowledge, this is the first human study to determine the effects of the RES on obese men with the MetS, under stable metabolic conditions in a randomized, placebo-controlled trial.

## 2. Materials and Methods

### 2.1 Subjects

Subjects were recruited from the surrounding community through advertisements in local newspapers and online and from the Rockefeller University subject repository. Eligible subjects were obese men (body mass index [BMI] 30–40 kg/cm2) with insulin resistance determined by euglycemic, hyperinsulinemic clamp (i.e., M ≤ 6.5 mg/kg/min), between the ages of 30–70 years, and three other features of the MetS. These features included fasting blood glucose ≥100 mg/dl, serum triglycerides >150 <500 mg/dl, high-density lipoprotein cholesterol ≤40 mg/dl, waist circumference ≥102 cm, and blood pressure ≥l30 mmHg systolic or ≥85 mmHg diastolic without medication, using the National Cholesterol Education Program American College of Physicians III 2005 criteria [21]. Exclusions were current smokers and subjects taking statins and medications metabolized by cytochrome p450 3A, elevated liver enzymes, diabetes (fasting blood glucose >125 mg/dl or hemoglobin A1C > 6.5%, or current diagnosis of diabetes), HIV infection, or abnormal thyroid function. Subjects gave a written informed consent using good clinical practice guidelines. The study was approved by the Institutional Review Board and the Advisory Committee for Clinical and Translational Science at The Rockefeller University (Protocol JWA-0786) and was registered with Clinical Trials.gov. # NCT01714102.

**Figure 1:**
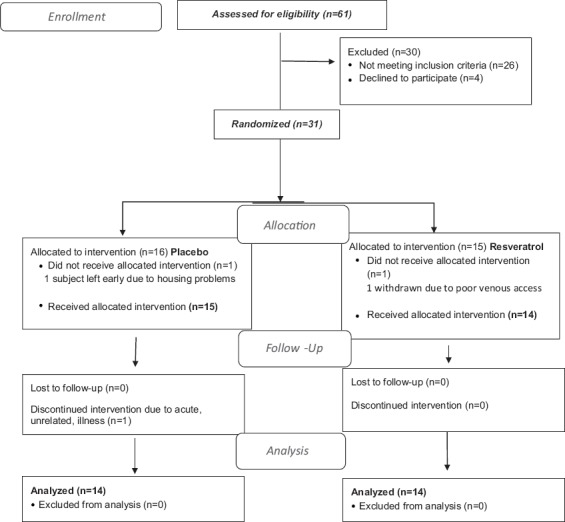
Trend flow chart.

Sixty-one subjects were screened; 31 met the enrollment criteria and were randomized 1:1 by the research pharmacist before entering the study (Figure 1). Due to the complexity and time required of the clamp procedure, the baseline clamp was performed as the final screen of the study, with subjects in the hospital to have it performed. Subjects understood, before enrolling, that they might be disqualified based on the result of the clamp. Withdrawn was one subject who left due to housing problems, one was withdrawn due to poor venous access, and one left due to unrelated illness. The remaining 28 subjects completed the 35-day study, 14 randomly assigned to each group (Figure 1). Eleven subjects were Caucasian and 17 were non-Caucasian (13 African Americans, 3 Afro-Latinos, and 1 African American/Asian). This single-center study was performed at The Rockefeller University Hospital (RUH) between October 2012 and September 2015.

### 2.2 Design and Setting

This was a double-blind pilot randomized parallel group design placebo-controlled study. Subjects initially were screened in the Outpatient Research Center at the RUH, where they met with the study principal investigator J.W. Screening procedures comprised of a complete history and physical examination, fasting blood testing, resting energy expenditure (REE), and waist measurements.

Eligible subjects were randomized by the research pharmacist in a 1:1 ratio using a web-based randomization program, to receive either RES or placebo. The study team and subjects were blinded as to the randomization to avoid bias. Subjects were admitted to the Rockefeller University inpatient metabolic unit for 35 days. They were fed for 4 days with an isocaloric western-style diet, (50% carbohydrate, 15% protein, 35% fat consisting of 13% saturated fat, 13% monosaturated fat, and 7% polyunsaturated fat) [22]. Calories required to keep weight stable were determined by the REE data, a 3 days’ food diary, and subject’s reported activity level. Developed by the Bionutrition Department, the diet included minimal amounts of RES-containing foods. On day 4, insulin sensitivity was measured by the euglycemic hyperinsulinemic clamp procedure. Insulin-resistant subjects (defined as M ≤ 6.5 mg/kg/min) completed the remaining baseline testing, which included blood tests (complete blood count, electrolytes, liver function tests, blood urea nitrogen, creatinine, and C-reactive protein), 75 g, 2-h oral glucose tolerance test (GTT), REE, waist measurement, daily BP monitoring, abdominal subcutaneous adipose tissue aspiration biopsy, fecal collection, and serum collection for insulin and lipids.

After baseline testing, subjects began receiving either two 500 mg Mega-RES 99% capsules (made exclusively from organically grown Japanese knotweed root) or two 500 mg placebo capsules, twice daily (Candlewood Stars, Danbury,CT). for 30 days, administered by the nursing staff. Only the research pharmacist was aware of the study drug allocation. The RES and placebo capsules were identical, placed in the medication cart by the pharmacist, and administered to the patients by registered nurses in identical packaging. Since RES is photosensitive [23], the product was stored in dark bottles. The measured RES content of the capsules was 524 mg each so that the total daily dose administered was actually 2096 mg. Body weight was monitored each morning in a hospital gown and maintained within 1.5% of baseline weight by adjusting caloric intake as needed. Subjects consumed an isocaloric, western-style diet, and no other foods or drinks were permitted. Subjects slept in the hospital where they always consumed breakfast and dinner and were provided with a packed lunch on days they did not have testing. On non-testing days, they were permitted to leave the hospital to work or to pursue other interests. Daily activity was monitored by a New Lifestyles NL-800 accelerometer (New Lifestyles, Lee’s Summit, MO). Safety blood laboratory tests and electrocardiogram testing were monitored. After 30 days of consuming the study drug or placebo, baseline testing was repeated.

### 2.3 Procedure methods

#### 2.3.1 Anthropometric measurements

Body weight was measured daily, using a Scale-Tronix 5002 scale (Welch Allyn, Skaneateles Falls, N.Y.) with a precision of ±0.1 kg. Subjects were weighed in a hospital gown, after an overnight fast and post-voiding. Height was measured at baseline with a Seca-216 stadiometer (Hamburg, Germany) in 1 mm increments. BMI was calculated as weight/height squared (kg/m2), using the National Institutes of Health (NIH) Standard Metric BMI calculator.

#### 2.3.2 Daily blood pressure monitoring

Manual blood pressure readings (Welch Allyn aneroid monitor, Skaneateles Falls, N.Y.) were taken by the hospital staff each morning and were recorded. The mean of the systolic and diastolic readings taken on the four mornings of baseline testing was statistically compared with the mean of the systolic and diastolic readings measured on the final 4 days of the study.

#### 2.3.3 REE

On days 7 and 35, REE was determined by indirect calorimetry using a Viasys Vmax Encore Calorimeter (Cardinal Health, Yorba Linda, Cal).

#### 2.3.4 2 h oral GTT

On days 6 and 34, a fasting 75 g oral glucose tolerance drink was administered, and serum glucose and insulin levels were determined at −10, −5, 0, 30, 60, 90, and 120 min. HOMA-IR scores were calculated from the 0-time point data: insulin (mU/l) X glucose (mg/dl).

#### 2.3.5 Abdominal fat biopsy aspiration

On days 7 and 35, subcutaneous abdominal aspiration biopsy of white adipose tissue was performed under local anesthesia, using a 4-mm liposuction needle, for gene expression analysis.

#### 2.3.6 Euglycemic hyperinsulinemic clamp

On days 4 and 32, euglycemic-hyperinsulinemic clamp was performed infusing 80 mU/m2/min Humulin R (Lilly, Indianapolis, IN). Glucose levels were monitored every 5 min using the YSI 2300 STAT Plus Glucose and Lactate Analyzer (Yellow Springs, OH.). Plasma glucose was maintained between 90 and 100 mg/dl by titration of intravenous 20% dextrose solution (Baxter Healthcare, Deerfield, Ill.) infusion.

#### 2.3.7 Fecal microbiome analysis

Fresh fecal samples were obtained in the hospital, from seven placebo-treated and nine RES-treated subjects, and samples were kept frozen at −80°C until analysis. Permission to study fecal samples in the remaining subjects could not be obtained.

Fecal DNA was extracted from 20 mg aliquots of feces using the PowerSoil DNA Isolation Kit (QIAGEN, Germantown, MD) according to the manufacturer’s protocol. Purified DNA from stool samples was amplified by PCR using the 16S rRNA V4-region primers (515F/806R) according to the Earth Microbiome Protocol. Amplified DNA samples were quantified using the PicoGreen Kit (Qiagen) and were pooled at equimolar ratios. Samples were then purified by PCR purification kit (Qiagen). Samples were multiplexed with unique barcodes and sequenced with 150-bp paired-end reads (2 × 150) on the MiSeq 2500 platform.

#### 2.3.8 Qiime analysis

The forward and reverse paired-end reads were joined using the *fastq-join* command from EA-utils, a command-line tool for processing biological sequencing data. Reads with a minimum overlap of 30 nucleotides (nt) and with perfect matching of bases between reads were demultiplexed and analyzed by quantitative insights into microbial ecology software (Qiime 1.9.1). Reads were filtered for base pairs with Phred score >20. The Phred score is a measure for base quality in DNA sequencing. The larger the Phred value, the better the quality of a sequenced base. The sequences were then clustered into operational taxonomic units (OTUs) using an open-reference approach with UCLUST, a high-performance clustering alignment and search algorithm capable of handling millions of sequences. These sequences were then referenced against the Greengenes 16S rRNA database (13_8 release), using Python Nearest Alignment Space Termination (PyNAST) tool. Analysis of variance (ANOVA) tests of OTU and genus-level abundances were performed in Qiime. A rarefaction analysis was accomplished using Chao-1 to estimate diversity (rare OTUs) from abundance data. Whole phylogenetic diversity (PD) was performed to measure alpha diversity. Unweighted UniFrac distances determined by the presence or absence of the species were calculated to assess beta diversity. The unweighted pair group method with arithmetic mean was performed for UniFrac-based jackknifed hierarchical clustering. Principal coordinate analysis was performed by calculating UniFrac distance matrices, and KiNG was used for graphical representation. Linear discriminant analysis effect size (LEfSe), a tool that can compare differences of relative abundance between two or more biological conditions, was used for analysis of the results [24]. Phylogenetic Investigation of Communities by Reconstruction of Unobserved States (PICRUSt) was used to predict the metagenomic content from the 16S rRNA sequencing data, and KEGG (Kyoto Encyclopedia of Genes and Genomes) pathway functions were categorized at level 3 [25].

#### 2.3.9 Plasma RES and its primary metabolite dihydroresveratrol (DHR) levels

These were determined in the laboratory of DSM Nutritional Products Limited, Kaiseraugst, Switzerland, before and after 30 days of RES administration. To determine free RES, an internal standard (RES-phenyl-13C6) was added to an aliquot of plasma followed by a liquid-liquid extraction. For conjugated RES forms, a digestion with β-glucuronidase was performed after addition of an internal standard, followed by liquid-liquid extraction. After centrifugation, an aliquot of the organic phase was evaporated to dryness, redissolved in injection solvent, and analyzed. Quantitation was achieved by the use of internal standards and an external standard calibration curve. Samples were analyzed by LC-MS/MS in negative electrospray mode and validated according to the bioanalytical method EMEA and FDA validation guidelines.

#### 2.3.10 Analysis of mega-RES 500 mg capsule

After extraction, RES was analyzed by RP-HPLC-UV applying an isocratic method with a phosphate buffer as mobile phase and the detection wavelength of 305 nm. Quantification was carried out using trans-RES as external standard, by a validated method.

#### 2.3.11 RNA sequencing of abdominal white adipose tissue

Abdominal subcutaneous adipose tissue biopsies were analyzed by RNA SeQ. Total RNA was extracted from approximately 0.5 g of frozen adipose tissue using a Qiagen RNeasy Lipid Tissue Mini Kit (Germantown, MD). RNA quality was assessed using an Agilent Bioanalyzer. Approximately 2–3 mg of RNA with RNA integrity number 0.7 was submitted. Twenty-one subjects consented to RNA sequencing of the adipose tissue, six did not give consent for analysis, and one subject did not undergo the biopsy due to anxiety. Adipose tissue from before and after treatment from 10 RES-treated and 11 placebo-treated subjects was referred for 50-bp paired-end readRNA sequencing polyA-enriched RNA at The Rockefeller University Genomics Research Center. Of those on RES, five were Caucasians and five were non-Caucasians. Gene expression was analyzed for pathway enrichment using Gene Set Enrichment Analysis (GSEA, Broad Institute, Cambridge MA) and Ingenuity Pathway Analysis (Qiagen).

The FASTQ files were first quality controlled through FastOC v0.11.15 [26]. Cutadapt v1.9.1 was used to locate and remove the adapter sequences from each high-throughput sequencing reads before mapping [27], as applied to trim the low-quality bases and TrueSeq adapters (times =2; quality base =33; quality cutoff =5; format =FASQT; and minimum length =25 –a AAAAAAAAAAAAATTTTTTTTTTTTT–a AGATCGGAAGAG– a CTCTTCCGATCT). Trimmed FASTQ files were aligned to the human genome (GRCH37) using STAR v.2.4.2 [28] aligner with default parameters. The alignment results were then evaluated through Qualimap v.2.2 [29], to ensure that all the samples had a consistent coverage, alignment rate, and no obvious 5’ or 3’ bias. Aligned reads were then summarized through feature Counts v1.5 [30]. The Ensembl gene annotation system, a web-based browser for vertebral genomes, was used for this purpose. The uniquely mapped reads (NH “tag” in bam file) that overlapped with an exon (feature) by at least 1 bp were counted, and then, the counts of all exons annotated to an Ensembl gene (meta-features) and were summed into a single number. Empirical analysis of digital gene expression (edgeR*)v.3.16.5 [31] was used to normalize the samples, and Voom (mean-variance modeling) from limma # v.3.30.11 was applied to estimate the differential log fold change in the expression of genes.

#### 2.3.12 Adipose tissue pathway enrichment analysis

A linear mixed-effect model as implemented in R limma package was applied to analyze differences in gene expressions from pre-to post-treatment. Moderated t-statistics were used for testing hypotheses about differential expression. The genes were ranked according to the magnitude of their significance, determined by the false discovery rate (FDR), p < 0.05. The ranked gene list was submitted to enrichment analysis in the GSEA software. The normalized enriched scores were tested for statistical significance and the gene sets with FDR < 0.05 were reported as significantly associated with changes from pre-to post-RES administration. This same procedure was used for a subgroup analysis that included only Caucasians.

## 3. Statistical Analysis

In this double-blind pilot randomized parallel group design placebo-controlled study, the one-sided hypothesis was that RES reduces insulin resistance in obese, weight-stable men with the MetS. The primary outcome was the euglycemic hyperinsulinemic clamp measured at day 30 of the study. Secondary outcomes included insulin resistance (GTT), systolic and diastolic BP, triglycerides, circulating inflammatory markers in serum and adipose tissue, and gene expression in adipose tissue measured at the same time points as primary outcome. In addition, the effects of RES on fecal microbiota composition and imputed metagenomic functions were determined.

### 3.1 Data protection and management

Unblinded, coded data were downloaded from a secure server, exported from REDCap to csv. format, and imported into IBM SPSS 19. Coded data and associated syntax and output files were maintained on a secure double-password protected desktop. Transfer of coded data was behind an institutional firewall through encrypted email. Less than 1% of data was found to be missing so that subsequent analyses proceeded.

### 3.2 Power analysis and sample size

Based on preliminary data from prior, unpublished studies, when randomization results in equivalent distribution of confounders across groups, the low variability of clamp results suggests extremely small sample sizes through the use of traditional power calculations. If one creates 14 matched pairs based on known risk factors, the risk of at least 10 of the more insulin-resistant subjects being in one group and at most five of the more insulin-resistant subjects in the other group (i.e., a 2:1 confounder ratio, or worse) is below 20% and the risk for a 1:5 ratio is below 5%. However, if there is more than one confounder and the confounders are at least partially independent, the resultant ratio may be less. Given the accuracy of the clamps and the exploratory nature of this study, a sample size of 14 subjects in each group provides balance for the comparison of study outcomes. Potential confounders and baseline data were found not to be significantly different between groups (Table 2). Randomization, therefore, resulted in homogeneous groups.

**Table 1:** Baseline metabolicsyndrome characteristics of subjects

Clinical Measurement	Placebo (n=14)		Resveratrol (n=14)		p

	Mean±SD	CI 95%	Mean±SD	CI 95%	
FBG (mg/dl)	104±22	92.–117	98±12	91–105	0.322

HDL (mg/dl)	42±9	37–47	40±7	36–44	0.672

TG (mg/dl)	146±6	114–179	125±75	82–168	0.409

SBP (mmHg)	119±9	115–124	118±6	115–121	0.717

DBP (mmHg)	77±6	74–81	78±2	77–80	0.457

Waist circ (cm)	116±8	111–120	117±11	111–123	0.813

Comparison of the baseline metabolic syndrome characteristics of the total group of resveratrol and placebo treated subjects. mg/dl: Milligrams per deciliter, FBG: Fasting blood glucose, HDL: High density lipids, TG: Triglycerides, SBP: Systolic blood pressure, DBP: Diastolic blood pressure, mmHg: Millimeters of mercury, cm: Centimeter, waist circ: Waist circumference, SD: Standard deviation, CI: Confidence interval.

**Table 2:** Baseline comparison of subjects by treatment

Clinical Measurement	Placebo (n=14)		Resveratrol (n=14)		p

	Mean±SD	CI 95%	Mean±SD	CI 95%	
Clamp M (mg/kg/min)	4.3±0.9	3.8–4.8	4.4±1.3	3.7–5.2	0.705

Insulin (mlU/L)	14+/8	9–19	22±20	10–33	0.194

HOMA (%)	3.7±2.3	2.3–5.0	5.5±6.3	1.8–9.1	0.322

120 min data 2 h GTT (mg/dl)	184±58	151–218	153±24	139–167	0.081

AUC (mg/dl)	345±77	301–389	310±41	286–333	0.145

WBC (k/mcl)	6.0±1.4	5.2–6.8	6.0±1.6	5.1–6.9	0.960

CRP (mg/dl)	0.33±0.17	0.24–0.43	0.58±0.34	0.39–0.78	0.734

REE (k/cal/day)	1778±204	1652–1898	1754±209	1627–1880	0.789

BMI (kg/m2)	33.8±3.3	32.0–35.7	35.0±3.0	33.2–36.7	0.317

Age (years)	47±8	42–52	48±9	43–60	0.772

Comparison of the baseline laboratory data, BMI and age of the total group of resveratrol and placebo treated subjects. Clamp M: mg/kg/min of dextrose required to maintain plasma glucose at level of 90–100 mg/dl during euglycemic hyperinsulinemic clamp; 120 min data: The blood glucose level at 120 min of a 2 h oral glucose tolerance test, GTT: Oral glucose tolerance test, AUC: Area under the curve, HOMA: Homeostatic model assessment, assesses insulin resistance and beta cell function, WBC: White blood cell count, CRP: C-reactive protein, TG: Serum triglycerides, REE: Resting energy expenditure, BMI: Body mass index, SD: Standard deviation, CI: Confidence interval.

### 3.3 Comparison of effects of the treatment groups on primary and secondary outcomes

The effects of the intervention on the primary and secondary outcomes were compared. The primary outcome of the analysis, the clamp data after 30 days, was compared between the two groups using a Wilcoxon/Mann–Whitney test. The remaining outcomes were tested for normal distribution within the groups, and independent sample two-tailed t-tests were conducted to test hypotheses that RES had a positive effect (i.e., reduction) on the primary and secondary outcomes.

### 3.4 Comparison within subjects of primary and secondary outcomes

Individual baseline data on the primary and secondary outcomes had been collected on all subjects allowing for estimates of changes from pre-to post-treatment within subjects, in addition to between the two treatment groups, on all outcome variables. Change scores were calculated for each subject on primary and secondary variables, using general linear models. Specifically, analyses of covariance (ANCOVA) were conducted on outcomes with premeasures of the same variable (e.g., baseline GTT 120 min time point, baseline clamp, and baseline mean systolic blood pressure). Group assignment was a fixed factor within the model.

### 3.5 Comparison of demographics on primary and secondary outcomes based on post-intervention outcomes

To examine the large within-group subject variance, individual patient trajectories were graphed over time. Subpatient populations were constructed blocked on race, ethnicity, and age. The means and variance for each subgroup were plotted and compared between groups for significance.

## 4. Results

### 4.1 Metabolic components

Of 61 subjects screened, 31 were found eligible and were entered into the study. Three subjects were withdrawn so that 28 subjects completed the study and 14 subjects were randomized to each group (Figure 1). The RES preparation was tolerated well, and measures of plasma RES levels demonstrated that RES subjects were compliant. At baseline, characteristic features of the MetS did not differ between the RES and placebo-treated groups (Table 1). Study subjects were all class 1-2 obese with similar ages and REE (Table 2). There was no evidence of any increase in inflammatory markers such as white blood count (WBC) and high sensitivity C-reactive protein (hsCRP).

Following 30 days of RES treatment, there were no significant differences in the measures that characterized the MetS between the RES and placebo-treated groups (Table 3A). The insulin clamp measure of insulin resistance did not differ between the RES and placebo treated nor did serum insulin and HOMA measures (Table 3B). However, the 120-min glucose concentration in the RES-treated subjects was significantly lower than in placebo subjects (p = 0.023), and the area under the curve for glucose levels during the GTT was marginally lower (p = 0.05). No other end point measures differed between the groups. With *a priori* significance level of 5%, differences between groups reached statistical significance on multiple outcomes when mean values were compared between groups (Table 4).

**Table 3A:** Posttreatment comparison of metabolic syndroe characteristics of resveratrol and placebo treated subjects

Clinical Measurement	Placebo (n=14)		Resveratrol (n=14)		p

	Mean±SD	CI 95%	Mean±SD	CI 95%	
FBG (mg/dl)	104±16	95–113	98±11	91.2–104	0.288

HDL (mg/dl)	41±8	37–46	40±8	36–45	0.664

TG (mg/dl)	130±50	101–159	138±86	86–188	0.762

SBP (mmHg)	120±13	113–128	123±13	116–131	0.476

DBP (mmHg)	79±6	75–82	81±5	78–84	0.207

Waist circ (cm)	115±9	110–121	116±10.4	110–122	0.860

FBG: Fasting blood glucose, HDL: High density lipids, TG: Triglycerides, SBP: Systolic blood pressure, DBP: Diastolic blood pressure, SD: Standard deviation, CI: Confidence interval.

**Table 3B:** Posttreatment comparison of resveratrol and placebo treated subjects

Clinical Measurement	Placebo (n=14)		Resveratrol (n=14)		p

	Mean±SD	CI 95%	Mean±SD	CI 95%	
Clamp M (mg/kg/min)	4.2±1.4	3.4–5.0	4.6±1.8	3.6–5.6	0.525

Insulin (mg/dl)	17±8	13–21	20±16	10–29	0.537

HOMA (%)	4.4±2.2	3.1–5.7	5.0±5.2	2.0–7.0	0.698

120 min data 2 h GTT (mg/dl)	172±39	150–195	139±13	120–158	0 0.023*

AUC (mg/dl)	346±64	309–383	304±42	280–328	0.050*

WBC (k/mcl)	5.9±1.3	5.1–6.7	5.2±1.5	4.3–6.1	0.209

CRP (mg/dl)	0.40±0.20	0 0.28–0.52	0.60±0.41	0.36–0.84	0.111

REE (k/cal/d)	1798±225	1662–1934	1778±228	1640–1976	0.821

BMI (kg/m2)	33.7±3.3	31.8–35.6	34.9±3.0	33.2–36.7	0.331

Comparison of the posttreatment metabolic syndrome characteristics (3A) and laboratory data and BMI (3B) for the total group of resveratrol and placebo treated subjects. *P≤ 0.05. GTT: Oral glucose tolerance test, AUC: Area under the curve, HOMA: Homeostatic model assessment, assesses insulin resistance and beta cell function, WBC: White blood cell count, CRP: C-reactive protein, TG: Serum triglycerides, REE: Resting energy expenditure, BMI:Body mass index, SD: Standard deviation, CI: Confidence interval.

**Table 4A:** Baseline comparisn of significant findings by subject race

Clinical Measurement	Caucasians (*n*=11)		Non-Caucasians (n=17)		p

	Mean±SD	CI 95%	Mean±SD	CI 95%	
Clamp M	3.9±1.1	3.2-4.7	4.6±1.1	4.1-5.2	0.090

120 min data 2 h GTT (mg/dl)	154±26	137-171	178±55	150-206	0.192

AUC (mg/dl)	318±33	295-340	334±77	294-373	0.518

GTT: Oral glucose tolerance test, AUC: Area under the curve, SD: Standard deviation, CI: Confidence interval.

**Table 4B:** Posttreatment comparison of significant findings by race within treatment groups

Clinical Measurement	Placebo (*n*=14)		Resveratrol (*n*=14)		p

	Mean±SD	CI 95%	Mean±SD	CI 95%	
	**Caucasians (*n*=6)**		**Caucasians (*n*=5)**		

Clamp M	4.3±1.5	2.7–5.9	4.9±2.5	1.7–8.0	<0.001*

120 min data 2 h GTT (mg/dl)	163±26	136–191	132±24	102–162	0.001*

AUC (mg/dl)	350±55	293–408	302±29	266–339	0.006*

	**Non-Caucasians (*n*=8)**		**Non-Caucasians (*n*=9)**		

Clamp M	4.2±1.5	3.0–5.4	4.5±1.4	3.4–5.5	0.940

120 min data 2 hGTT (mg/dl)	179±47	140–218	143±38	114–173	0.962

AUC (mg/dl)	344±75	281–405	305±49	267–343	0.376

Comparison of the baseline (4A) and posttreatment (4B) glucose tolerance and clamp M data in the Caucasian and noncaucasian group of resveratrol and placebo treated subjects. GTT: Oral glucose tolerance test, AUC: Area under the curve, SD: Standard deviation, CI: Confidence interval.

In a *post hoc* ANCOVA to control for baseline data (Table 4A), we observed a difference between the treatment groups (RES vs. placebo) when divided into Caucasian and non-Caucasian subjects, in the effects of RES on glucose homeostasis and race. As shown in Table 4B, there were highly significant effects on insulin resistance as measured by the insulin clamp technique in Caucasian when compared to non-Caucasian subjects (p < 0.001). Furthermore, the 120-min end point glucose level and the glucose concentration area under the curve during the GTT were significantly lower in Caucasian subjects (Table 4B). At baseline, these two groups did not differ in age, BMI, or the number of components of the MetS (Table 5).

**Table 5:** Baseline comparison by race in resveratrol treated subjects (*n*=14)

Clinical Measurement	Caucasians (*n*=5)		Non-Caucasians (*n*=9)		p

	Mean±SD	CI 95%	Mean±SD	CI 95%	
FBG (mg/dl)	95±10	83.0–107	102±16	90–114	0.366

HDL (mg/dl)	37±3	33–40	41±6	37–46	0.136

TG (mg/dl)	177±100	52–301	101±38	72–130	0.170

SBP (mmHg)	117±5	110–123	119±6	114–124	0.415

DBP (mmHg)	78±3	74–82	80±2	78–81	0.233

Waist circ (cm)	121±12	108–135	115±10	107–122	0.258

BMI (kg/m2)	34.1±2.8	30.6–37.6	35.6±2.9	33.3–37.8	0.375

Age (years)	49±9	38–59	47±9	41–54	0.785

Comparison of the baseline characteristics and laboratory data of the caucasian and noncaucasian resveratrol treated subjects. FBG: Fasting blood glucose, HDL: High density lipids, TG: Triglycerides, SBP: Systolic blood pressure, DBP: Diastolic blood pressure, SD: Standard deviation, CI: Confidence interval, BMI: Body mass index.

Since these group differences in glucose homeostasis could have resulted from differences in RES absorption, distribution, or metabolism, we measured fasting level of RES and its primary metabolite, DHR, in all study subjects. The overall data in the total of 14 RES treated subjects varied greatly between subjects (Table 6). There were no differences between Caucasian and non-Caucasian subjects in plasma concentrations of RES and DHR. We also determined potential correlations between plasma concentrations of RES and DHR and the MetS components, adipose tissue gene expression, and fecal microbiota and found no significant correlations (data were not shown).

**Table 6:** Plasma resveratrol and dihydroresveratrol levels post resveratrol treatment (*n*=14)

Components	Mean±SD		p

	Caucasians (*n*=5)	Non-Caucasians (*n*=9)	
RES (nmol/L)	23,203±8,467	27,349±13,908	0.559

DHR (nmol/L)	2,276±3,411	5,709±10,111	0.482

Terms: RES nmol/L: Resveratrol measured in nano-moles/liter. DHR nmol/L: Dihydroresveratrol, the primary metabolite of resveratrol, measured in nano-moles/liter. RES: Resveratrol, DHR: Dihydroresveratrol, SD: Standard deviation.

### 4.2 Adipose tissue

To determine changes with RES treatment that may influence glucose metabolism, abdominal subcutaneous adipose tissue biopsies were analyzed by RNA SeQ and GSEA enrichment analysis. After correction for multiple comparisons, no significant changes in individual gene expression and gene expression pathways between biopsies taken before and after the study in placebo-treated subjects were detected. Correcting for multiple comparisons, there also were no significant differences between RES and placebo-treated subjects at the end of the study.

To gain further insight into possible effects of RES intervention on adipose tissue gene expression for subsequent, more detailed study, we evaluated a selection of the top individual genes that were altered in the RES intervention group of subjects (Table 7). Upregulated gene expression occurred with the RAB 40A member of the RAS oncogene family, microRNA 192, apolipoprotein C4, a growth differentiation factor, a member of the TNF 2 receptor superfamily, the 5-hydroxytryptamine receptor1B, two olfactory receptors, and 32 long, intergenic non-protein coding RNAs. We found no significantly downregulated genes by RES treatment. Contrary to expectations, genes related to SIRT1 and mTOR pathways did not change.

**Table 7:** Postresveratrol selection of altered genes in subcutaneous adipose tissue

Gene symbol	Gene designation	p	Log fold change
RAB40A	RAB40A member RAS oncogene family	0.00013	0.2715

FAM230C	Family with sequence similarity 230 member C	0.00026	0.2330

MIR192	micro-RNA 192	0.0012	0.2117

APOC4	Apolipoprotein C	0.0014	0.1934

MFAP2	Microfibrillar associated protein 2	0.0019	0.2434

GDF2	Growth differentiation factor 2	0.0027	0.2564

OR52E4	Olfactory receptor family 52, subfamily E, member 4	0.0031	0.1918

OR4D6	Olfactory family 4, subfamily D, member 6	0.0033	0.1820

TNFRSF13B	TNF receptor superfamily member 13B	0.0036	0.2052

HTR1B	5-hydroxytryptamine receptor 1B	0.004	0.2128

LINC00982	Long intergenic non-protein coding RNA 982	0.005	0.5994

LINC01356	Long intergenic non-protein coding RNA 1356	0.005	0.5141

Significantly altered gene expression data in adipose tissue following resveratrol treatment.

By GSEA analysis, the three Caucasian subjects were negatively enriched with the TGF beta signaling gene expression pathway compared to the five non-Caucasians from whom adipose tissues were available for analysis.

### 4.3 Comparison of intestinal bacterial communities in the placebo and RES treatment groups

**Figure 2:**
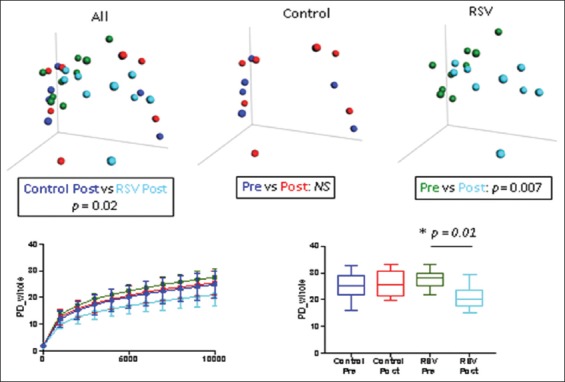
Differences in fecal microbiota between the resveratrol (RES) and placebo-control total subject groups. Upper panels: β-diversity as determined by unweighted UniFrac analysis; Lower panels: Differences in α-diversity between groups, left, mean ± standard deviation; right, Median + interquartile range box and whisker (95%) confidence interval. Plot color codes: Blue: Placebo-control pre-treatment; Red: Placebo-control post-treatment; Green: RES pre-treatment; Light blue: RES post-treatment. In RES-treated subjects, significant changes were seen in alpha-diversity, as measured by phylogenetic diversity (p = 0.01), and community structure, as measured by beta diversity (p = 0.007). There were no changes in the placebo group pre-and post-treatment.

Fecal samples were available for the study from seven Caucasian and nine non-Caucasian subjects. Nine subjects (3 Caucasians and 6 non-Caucasians) received RES. In these subjects, both alpha-diversity as measured by PD and community structure as measured by beta diversity changed significantly over the course of the study, but as expected, no significant differences developed in the placebo-treated controls (Figure 2). The community structure did not differ significantly pre-treatment between the two groups but differed post-treatment (p = 0.01). The data showed that species richness (alpha-diversity, as measured by PD) fell over the course of the study in the total of 16 subjects in whom specimens were available but had no significant differences in the community structure (beta differences as measured by UniFrac distances).

**Figure 3:**
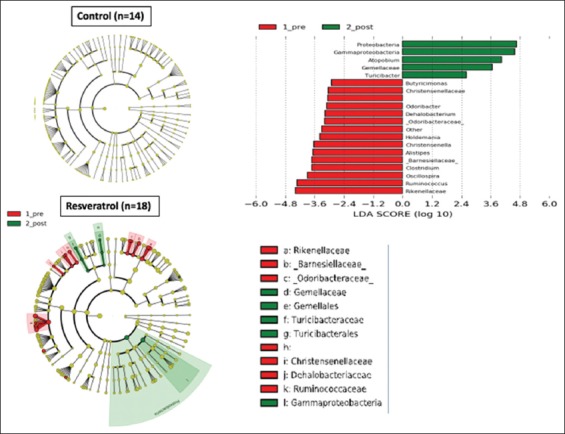
LefSe Cladogram and left anterior descending score of treatment differences. Identification of significant taxonomic differences associated with the total placebo-control group (n = 14 samples) and of the total resveratrol (RES) group (red: Pre-treatment, green: Post-treatment) (n = 18 samples). (Right) histogram of the linear discriminant analysis scores was calculated for the most differential taxa with RES treatment.

Next, we asked whether individual taxa showed significant changes in relative abundance in the RES and the placebo groups (Figure 3). In placebo controls, there were no significant differences in the taxa comparing the pre-and post-treatment specimens. However, several taxa changed significantly in the RES subjects with a fall in *Rikenellaceae*, *Ruminococcus, Oscillospira*, *Clostridium*, *Alistipes, Odoribacter*, and *Butyricimonas* and a rise in *Gammaproteobacteria, Gemellaceae*, *Turicibacter*, and *Atopobium*.

**Figure 4:**
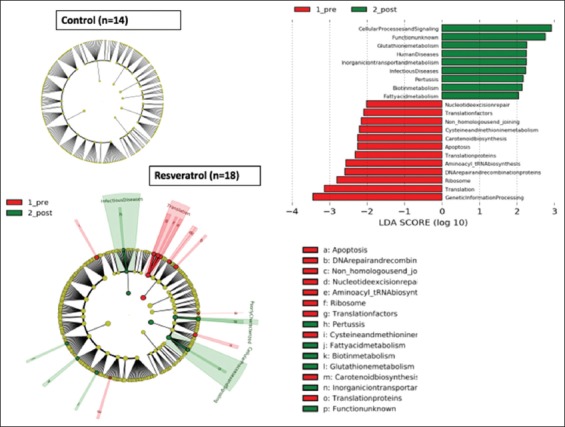
Phylogenetic Investigation of Communities by Reconstruction of Unobserved States (PIECRUSt) functional analysis of the control (placebo) and resveratrol (RES) treatments in the total subject group. Analyses using functions imputed by the PICRUSt algorithm, with score of linear discriminant analysis [LDA] >2. Histogram of the LDA scores was calculated for the most abundant taxa altered by the RES treatment.

In the PICRUSt comparative analysis of imputed biochemical pathways in the metagenome (Figure 4), placebo-treated subjects again showed no significant differences, whereas the RES group showed decreases in pathways related to ribosomal translation, aminoacyl tRNA biosynthesis, apoptosis, carotenoid biosynthesis, and cysteine and methionine metabolism. Post-RES treatment, the dominant pathways were glutathione metabolism, ion transport and metabolism, pertussis, biotin metabolism, and fatty acid metabolism.

Since we found several differences in glucose tolerance and insulin resistance between the Caucasian and non-Caucasian RES-treated subjects, we examined changes in fecal microbiota corresponding to these individuals. There were no significant differences in either alpha or beta diversity between Caucasian and non-Caucasian subjects (data are not shown). However, LefSe showed significant taxonomic differences between these two groups. In the Caucasians, *Alistipes, Collinsella, Christensenella, Holdemania, and Turicibacter* were among the taxa that fell during the treatment and *Bilophila* rose (Appendix Figure F1). In non-Caucasians, *Ruminococcaceae*, *Alphaproteobacteria, Christensenella, Odoribacter*, and *Clostridium* were taxa that fell during treatment, while *Proteobacteria* rose (Appendix Figure F2.). In pre-study Caucasians, there was significant over-representation of *Collinsella, Clostridiaceae*, and *Ruminococcus*, but *Streptococcus* and *Lactobacillales* were overrepresented in the non-Caucasians (Appendix Figure F3). Post-treatment, the Caucasian subjects showed significantly higher levels of *Akkermansia muciniphila*, *Fusobacteria*, and *Megamonas*, compared to the non-Caucasians (Appendix Figure F4).

## 5. Discussion

This double-blind, placebo-controlled study of a large dose of RES (2 g daily) did not show a significant effect on insulin sensitivity or glucose homeostasis, by our primary endpoint of euglycemic hyperinsulinemic clamp, when compared to the placebo-treated group of subjects. Glucose homeostasis was marginally affected as measured by GTT. The placebo and RES groups did not differ in factors contributing to the MetS or other potentially important end point variables. Our essentially inconclusive results agree with a 5-week cross-over study of RES 1 g/day in diet-controlled diabetic subjects [32]. Furthermore, this is consistent with a placebo-controlled study of RES 150 mg or 1 g/day in moderately obese but otherwise healthy community-living men [33], as well as a study by Poulsen et al. [34], which used RES 1.5 g/day in obese subjects and failed to improve insulin resistance.

These studies were in contrast with earlier studies in which RES 1–2 g/day was provided to older subjects with impaired glucose tolerance who showed modest improvement in insulin resistance estimated from the Matsuda index [11] and a 3-month open-label study of RES 250 mg/day in patients with type 2 diabetes taking oral antidiabetic medications [35]. Crandall, in an editorial, wondered whether RES would be effective only when glucose homeostasis was clinically impaired [36]. Korshalm et al. [37] agreed that subjects with modest insulin resistance are optimal to measure effects of RES on insulin resistance, but that healthy individuals with normal glucose homeostasis might not be affected by RES. In addition, longer studies may be necessary to determine the effect of RES on chronic conditions such as insulin resistance and low-grade inflammation.

It has been stated that there is sufficient information from rodent studies that RES has the capacity to improve insulin sensitivity and reduce blood glucose levels accompanied by modulation of inflammatory response to various stimuli. RES has been used in conjunction with metformin to improve insulin sensitivity and glucose homeostasis in pre-diabetic subjects [38]. However, studies in humans are fragmentary and generally inadequate [39].

Unexpectedly, when we performed a post-hoc analysis of the several factors that could have influenced our data, including the effects of race, the Caucasian subjects showed a highly significant effect of RES on glucose homeostasis, with reduction in insulin resistance, and a decline in both the 120-minute glucose concentration and the area under the curve in the oral glucose tolerance test, whereas non-Caucasian subjects had no significant effects on these measures. However, the dispersion of the non-Caucasian subjects’ 120-minute glucose concentration and the area under the curve was greater than the Caucasian subjects, which could decrease the likelihood of significant finding within the non-Caucasian subjects.

These differences could have reflected variation in RES absorption, known to be inconsistent [16], or in RES distribution and metabolism. Our current data on fasting plasma concentration of RES and its primary metabolite DHR did not explain the difference in glucose homeostasis with RES administration in the Caucasian subjects. We recognize that our study was powered for our primary outcome, a change in insulin resistance, and not for the secondary outcomes. This poses the potential that a type 2 error might have occurred in the analysis of the secondary outcomes.

Our data are in contrast with a Dutch comprehensive metabolic study of 11 grade 1 obese healthy men without MetS and mean 26% body fat, who were administered RES in a dose of 150 mg/day for 30 days in a placebo-controlled, cross-over study [40]. These authors reported that RES reduced resting metabolic rate, muscle inflammation, and intramyocellular lipid content. The RES-treated group in this study also showed reductions in fasting serum glucose, triglycerides, leptin, insulin, and HOMA, as well as several circulating markers of inflammation. The reasons for the differences between our results and their data are unclear. It is of interest that the subjects in the Timmers study were all Caucasians (personal communication), and thus, their improvement with RES may have paralleled the data in our Caucasian subjects. The RES dose of 150 mg/day and the fasting plasma levels of RES and DHR in the Timmers’ study were much lower than those measured in our study. Lower concentration effects might reflect an inverted U effect. Further studies of RES administration to obese or MetS Caucasians and non-Caucasian subjects seem warranted.

We included studies of subcutaneous white adipose tissue gene expression, since adipose tissue dysfunction is common in obese subjects, and RES might improve inflammation in fat tissues. Although we found no significant changes in the expression of genes and gene pathways in adipose tissue after correction for multiple factors, the selection of the individual genes that were most upregulated by RES included two olfactory genes. Several olfactory receptor genes in adipose tissue have been reported to modulate increased fat mass in obesity [41]. In rodents fed a high fat controlled diet, RES 30 mg/kg body weight was shown to reduce body weight, fat mass, and white adipose tissue lipolytic activity [42]. Others have described downregulation of the mTOR pathway improving glucose and lipid metabolism in mice [10,20], and inflammatory markers [19], after RES administration. These data differ from our null findings in human subcutaneous adipose tissue. In contrast, in another human study, RES at a dose of 150 mg/day for 30 days in obese men was reported to reduce adipocyte size associated with regulation of genes linked with cell cycle regulation and lysosomal activity, suggesting stimulation of an alternative pathway of lipid metabolism [43]. The reasons for the discrepancy between the two studies are unclear, but these data were taken from subjects in the cross-over metabolic study by Timmers discussed previously.

Increasing evidence has focused on the role of the gut microbiota in determining the metabolism of drugs and certain over-the-counter supplements [44]. Furthermore, drugs and supplements can alter the composition and the function of the microbiota in the gut [45]. Although RES has been shown to affect the gut microbiome *in vitro* and in rodents *in vivo* [46], these data are not consistent across studies. A comprehensive metabolomic analysis on blood, urine, adipose tissue, and skeletal muscle tissue in middle-aged men with MetS randomized to receive either RES or placebo treatment for 4 months found that the composition of the gut microbiota was altered by RES treatment, with a reduction in sulfated androgen precursors in blood, adipose tissue, and muscle, increasing these metabolites in the urine [37].

The microbiome may also affect the metabolism of RES [47]. The principal metabolite is DHR, but further molecular changes include glucuronidation and sulfation [46], which occur in the gastrointestinal tract and liver. A detailed *in vitro* study of the effects of the gut microbiota on RES showed that the major RES metabolite resulting from gut microbial action was DHR and indoxy metabolites, including the formation of lumularin [16].

In the panel of microbes used for these *in vitro* studies, *Slakia equolifaciens* and *Adlercreutzia equolifaciens* were the principle producers of DHR. In mice, RES 200mg/kg/day was shown to improve gut microbial dysbiosis as measured by increased *Lactobacillus* and *Bifidobacterium* species and reduced *Enterococcus faecalis* [17]. *Lachnospiraceae* often produces butyrate, a beneficial short-chain fatty acid [48]. By inhibition of several commensal microbiota, RES can attenuate the gut microbial metabolism of TMAO [18]. Indeed, in TMAO-fed Apo-E -/-mice, RES inhibited atheroma formation through changes in such gut microbiota accompanied by increased bile salt hydrolase deconjugation of conjugated bile salt acids.

In our study, RES reduced microbial diversity and led to several changes in individual microbiota. RES-treated Caucasian subjects who showed improvement in insulin sensitivity and glucose homeostasis also had a significant increase in *A. muciniphila*, a microbe that, in experimental animals, has been inversely associated with obesity, diabetes, and low-grade inflammation [49]. Administration of a purified *A. muciniphila* membrane protein to mice also has improved insulin sensitivity and reduced fat mass [50], consistent with potential therapeutic efficacy.

Although other investigators have focused on the role of RES as a SIRT activator, we found no change in SIRT gene expression in adipose tissue. SIRT1 has been linked to both lipid and glucose homeostasis. In white adipose tissue, SIRT1 inhibited adipogenesis in precursor cells and reduced fat storage in differentiated cells [51]. SIRT1 can also regulate glucose homeostasis by altering different tissue targets. In pancreatic β-cells, SIRT1 is a positive regulator of insulin secretion and can protect β-cells against oxidative stress by deacetylation of FOX-0 proteins. In the liver, SIRT1 may regulate gluconeogenesis. In target tissue, SIRT1 may affect glucose homeostasis by modifying the responses of target cells to insulin, by regulating the activity of PGC-1α [52].

## Conclusions

We demonstrated only minor overall effects of the administration of RES in a dose of 2 g/day to obese men with insulin resistance and the MetS. However, *post-hoc* analysis showed a significant improvement in insulin sensitivity and glucose tolerance during a 2-h oral GTT, in RES-treated Caucasian subjects. These improvements were not seen in non-Caucasians treated with RES. This observation was not explained by differences in RES absorption or metabolism as determined by measuring plasma concentrations of RES and its primary metabolite DHR or by examining the effects on adipose tissue gene expression. In fecal microbiome, RES altered alpha and beta diversity in treated subjects compared to placebo control subjects. Further studies of RES in Caucasian and non-Caucasian insulin resistant, obese subjects are needed to confirm our finding and to seek functional explanation for these differences.

## Conflicts of Interest Disclosure

The authors declare no conflicts of interest.
